# Genetic Diversity of *Echinococcus granulosus* in Southwest China Determined by the Mitochondrial NADH Dehydrogenase Subunit 2 Gene

**DOI:** 10.1155/2014/867839

**Published:** 2014-01-30

**Authors:** Jiahai Wang, Ning Wang, Dandan Hu, Xiuqin Zhong, Shuxian Wang, Xiaobin Gu, Xuerong Peng, Guangyou Yang

**Affiliations:** ^1^Department of Parasitology, College of Veterinary Medicine, Sichuan Agricultural University, Ya'an 625014, China; ^2^Department of Chemistry, College of Life and Science, Sichuan Agricultural University, Ya'an 625014, China

## Abstract

We evaluated genetic diversity and structure of *Echinococcus granulosus* by analyzing the complete mitochondrial NADH dehydrogenase subunit 2 (*ND2*) gene in 51 isolates of *E. granulosus sensu stricto* metacestodes collected at three locations in this region. We detected 19 haplotypes, which formed a distinct clade with the standard sheep strain (G1). Hence, all 51 isolates were identified as *E. granulosus sensu stricto* (G1–G3). Genetic relationships among haplotypes were not associated with geographical divisions, and fixation indices (Fst) among sampling localities were low. Hence, regional populations of *E. granulosus* in the southwest China are not differentiated, as gene flow among them remains high. This information is important for formulating unified region-wide prevention and control measures. We found large negative Fu's Fs and Tajima's *D* values and a unimodal mismatch distribution, indicating that the population has undergone a demographic expansion. We observed high genetic diversity among the *E. granulosus s. s.* isolates, indicating that the parasite population in this important bioregion is genetically robust and likely to survive and spread. The data from this study will prove valuable for future studies focusing on improving diagnosis and prevention methods and developing robust control strategies.

## 1. Introduction

Cystic echinococcosis (hydatid disease) is an important and globally distributed parasitic zoonosis caused by the larval stage of the cestode parasite *Echinococcus granulosus *complex [1]. Intermediate hosts, which include humans, sheep, goats, cattle, yak, camels, and other wild mammals, become infected by ingesting the parasite's eggs from infected carnivores (the definitive hosts). Subsequently, a larval stage (metacestode) develops as a cyst in the internal organs (mainly in liver and lungs) of the intermediate host.

The causative agent of cystic echinococcosis was traditionally regarded to be a single species, *E. granulosus*. However, recent research has shown that *E. granulosus* is a species complex consisting of several taxa that differ in adult morphology, their preferences for intermediate hosts, and their pathogenicity to animals and humans [2]. But now, this species complex is differentiated into ten genotypes (G1–G10) [3–8]. Moreover, some researchers have suggested that* E. granulosus* should be classified as four species based on the substantial molecular differences in both mitochondrial and nuclear DNA genes: *E. granulosus sensu stricto* (genotypes G1–G3), *E. equinus *(genotype G4), *E. ortleppi *(genotype G5), and *E. canadensis *(genotypes G6–G10) [9], though the status of *E. canadensis* is still disputed [9–12]. Meanwhile, a new independent taxon named *E. felidis* (lion strain) was isolated from South Africa [13].

In China, cystic echinococcosis has been reported in more than twenty provinces and is particularly prevalent [14, 15]. However, to date, infections have been ascribed to just two *E. granulosus* genotypes; G1 (a sheep strain) and G6 (a camel strain) [16] Southwest China is one of the most serious areas of* E. granulosus* infections in China. The past geologic events and climate fluctuations lead to a high biodiversity of species in this area [17, 18]. In addition, *E. shiquicus*, a new species of *Echinococcus*, has been recently discovered in this region [19]. Recently, the first human CE case infected with G5 genotype (cattle strain) in Asia has been reported [20]. For these reasons, it is critical to understand the genetic composition and structure of the *E. granulosus *complex in this region. In this study, we provide the first investigation of the molecular diagnostics of cystic echinococcosis infections in Southwest China.

Mitochondrial DNA has been widely used in population genetics to elucidate phylogenies, as it experiences high mutation and low recombination rates and thus best reflects population genetic structure, population differentiation, and species relationships [21]. The NADH dehydrogenase subunit 2 gene (*ND2* gene) evolves faster than other mitochondrial genes and is widely applied in molecular systematics and population genetics studies [22–25]. We used the *ND2* gene as a genetic marker to investigate the genetic diversity and structure of *Echinococcus granulosus* within Southwest China. This information will be essential for further studies investigating the biology and transmission dynamics of these parasites, especially to humans, and will underpin research on the diagnosis, control, and prevention of this disease [2, 26–29].

## 2. Materials and Methods

### 2.1. Sample Collection

Larval isolates of *E. granulosus* (*n* = 51) were collected from three intermediate host species (sheep, yak, and humans) in three locations in Southwest China (Qinghai, Sichuan, and Tibet; Table 1).

### 2.2. PCR Amplification, Purification, and Sequencing

Total DNA was extracted using standard phenol-chloroform techniques [30] and then stored at −20°C. The complete *ND2* gene was amplified using primers (P1: 5′-ATTGGACATTGTGTCTAGG-3′ and P2: 5′-GTTACTCCCATCAATGAGA-3′) that were designed based on the G1 genotype of *E. granulosus *(AF297617). The PCR mixture was prepared in a final volume of 25 *μ*L containing 1 *μ*L of template DNA, 1 *μ*L of each primer, 12.5 *μ*L of 2 × *Taq* PCR Master Mix, and 9.5 *μ*L of the reaction buffer supplied by the CoWin Company (Beijing). Thermal cycling was performed with initial denaturation for 4 min at 94°C followed by 35 cycles of 50 s at 94°C, 45 s at 48°C, 50 s at 72°C, and a final extension of 10 min at 72°C. PCR products were sequenced three times by the Invitrogen Trading Company (Shanghai).

### 2.3. Data Analysis

The DNAMAN program was used to align and compare the reference sequences to the nucleotide sequences identified in our study (Table 2). The percentage divergence of nucleotide sequences was determined using MEGA 5.0 [31] applying Kimura's two-parameter model with a *γ*-shaped parameter (alpha = 0.05) [32]. The maximum likelihood tree was also constructed using MEGA 5.0 [31] applying Kimura's two-parameter model with 1000 bootstrap replications. Bayesian phylogenetic analyses were performed and tested using MrBayes version 3.1.2 [33]. We used TCS version 1.21 to construct networks based on the criterion of statistical parsimony [34]. The Arlequin package (version 3.5.1.2) was employed to calculated genetic diversity indices (number of haplotypes, haplotype diversity, and nucleotide diversity) [35]. Pairwise fixation indices (Fst), which estimate the degree of gene flow between two populations, were calculated with the Arlequin package. Mismatch distributions were used to test for demographic signatures of population expansions within mtDNA lineages [36]. The neutrality indices of Tajima's D and Fu's Fs were calculated using the population genetics package Arlequin [37, 38].

## 3. Results

### 3.1. Variations in Nucleotide Sequences

All 51 isolates were successfully amplified for the complete *ND2* mitochondrial gene (882 sites; similarity with *E. granulosus* G1 [AF297617] was 96.71% to 99.89%). Base frequencies were unequal (A = 0.1634, T = 0.5159, C = 0.0745, and G = 0.2.462), and an anti-G bias was detected, which is characteristic of the mitochondrial genome (the frequency of A + T [67.93%] was higher than the frequency of G + C [32.07%]) [39]. Overall, there were 52 point mutations, of which 34 were parsimony informative and 17 single-point mutations. We obtained 39 transition mutations and 16 transversion mutations (Table 3). No deletions and indel sites were obtained.

### 3.2. Phylogenetic Analyses and Genotyping

**Figure 1:**
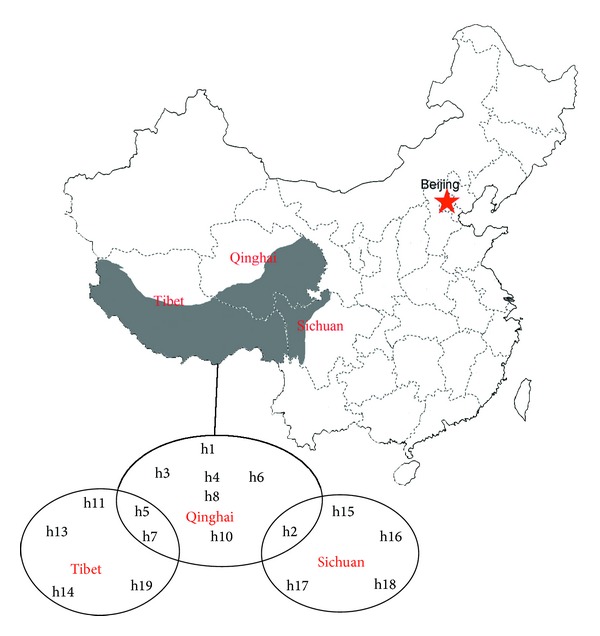
The localities of *E. granulosus* mitochondrial NADH dehydrogenase subunit 2 (*ND2*) haplotypes. The gray part of map is Southwest China.

**Figure 2:**
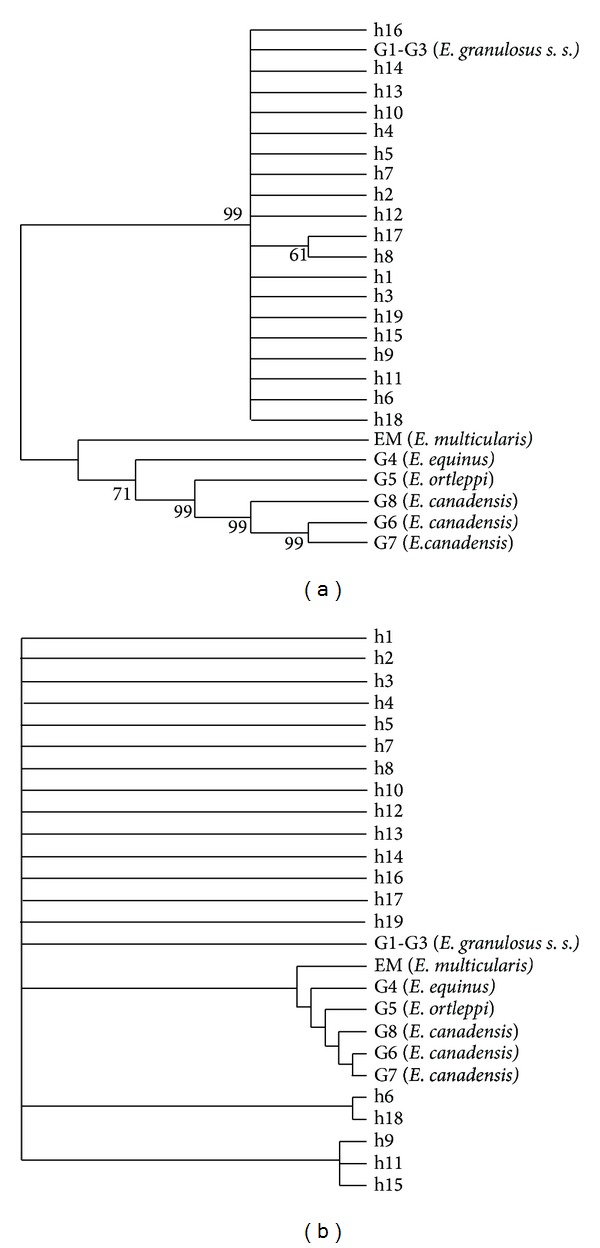
(a) Phylogenetic relationships among the 19 haplotypes inferred by maximum-likelihood methods. (b) Phylogenetic relationships among the three populations of the *E. granulosus* haplotypes based on the *ND2* gene inferred by Bayesian methods.

We detected 19 mtDNA haplotypes (labeled H1 to H19; GenBank ID: KC897670-KC897688) within the 51 isolates (the localities of the haplotypes are shown in Figure 1). The maximum-likelihood phylogram clearly showed that *ND2 *formed one clade and was not divided into regional clades according to the allopatric distributions of the isolates (Figure 2(a)). All 19 haplotypes grouped with the G1–G3 genotype in a large clade, which was distinct from the other strains. Accordingly, all 51 isolates were classified as *E. granulosus sensu stricto* (genotypes G1–G3). Results from the Bayesian tree analysis depicted a similar topology to the maximum-likelihood phylogram (Figure 2(b)).

### 3.3. Genetic Polymorphism Analysis and Population Expansion

The overall haplotype diversity of *E. granulosus *in Southwest China was high (hd = 0.898 > 0.5%), although nucleotide diversity was low (Pi = 0.005 < 5%). The genetic distance between haplotypes (Kimura 2-parameters) ranged from 0.001 to 0.028 (average genetic distance = 0.007; Table 4). Results of AMOVA showed that the majority of the variation existed within regions/populations, because there was 90.73% variation within the Southwest China population and 9.27% variation of *E. granulosus s. s.* among Southwest China subpopulations (Table 5).

**Figure 3:**
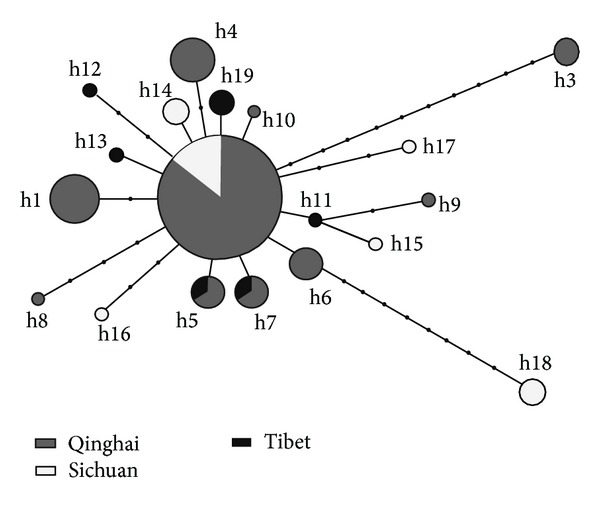
Haplotype network for the three sampling localities of *E. granulosus*. Each haplotype is represented by a circle. Circle sizes are proportional to the corresponding haplotype frequencies. Small black dots represent hypothetical missing or unsampled ancestral haplotypes.

Assuming that the ancestral haplotype is still present in the population, statistical parsimony networks were constructed, in order to discern the genealogical relationship among the haplotypes. Haplotypes of different regions were mixed together and there was no relationship between haplotype affinities and their locations (Figure 3). However, the resulting network showed a star-like expansion, with one common ancestral haplotype (H2) occupying the center of the network (Figure 3). There were one to ten mutational steps between the ancestor and the other haplotypes.

**Figure 4:**
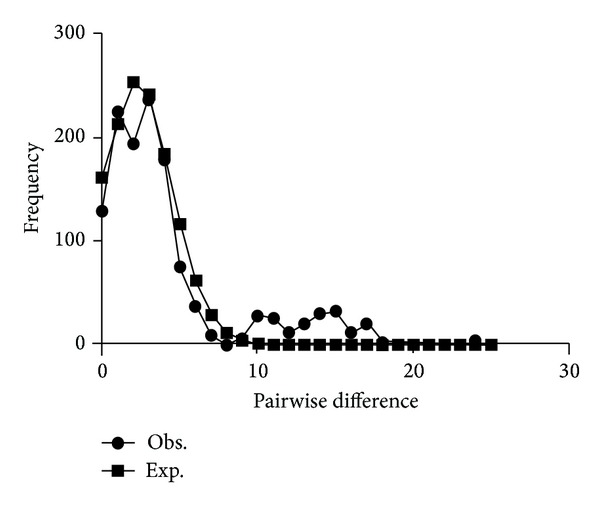
Mismatch distributions of the *ND2* haplotypes of *E. granulosus* in Southwest China.

Fst values among the three sampling regions of Southwest China were low and ranged from 0.037 to 0.143 (Table 6), indicating that the geographical populations were not genetically differentiated from one another. However, based on the significantly large negative Fu's Fs and Tajima's *D* values (Table 4), we can infer that the population of *E. granulosus *in Southwest China has undergone a demographic expansion. The unimodal distribution of the mismatch distribution supports this hypothesis of a sudden-expansion model (Figure 4).

## 4. Discussion

Southwest China is becoming a model region for biodiversity research and a major area for *echinococcosis* within China. This study used *ND2* gene to investigate the molecular systematics of *E. granulosus* in this region for the first time and revealed there to be considerable genetic diversity within species complex within this region but no evidence of complete population differentiation.

We observed a distinct anti-G bias in the nucleotide sequences of the *ND2* gene of *E. granulosus*. A similar result was found for other *Echinococcus* genes by Nakao et al. [40]. Variable sites occurred mainly in the third codon position, while the second codon position exhibited the least variation, supporting observations that the third codon position of mitochondrial protein genes evolves fastest, while the second codon position evolves slowest [41]. We obtained 39 transitions and 16 transversions (a transitions/transversions ratio of 2.4 > 2.0), indicating that the mutations of mitochondria *ND2* gene of *E. granulosus* are not saturated and are suitable for the analysis of genetic variation [42].

Of the 19 haplotypes we defined (from the 51 *E. granulosus *isolates), one (H2) was shared between the Qinghai and Sichuan populations and two (H5 and H7) were shared between the Qinghai and Tibet populations, while no haplotypes were shared between the Sichuan and Tibet populations. The haplotype phylogenetic tree showed that *E. multilocularis* was genetically distinct from *E. granulosus s. s.* (G1, G4, G5, G6, G7, and G8) and that all 19 haplotypes (and thus all isolates) grouped with the standard sheep strain (G1) and were thus classified as *E. granulosus s. s.* (genotypes G1–G3). This finding is similar to previous studies that found that *E. granulosus s. s.* (G1–G3) is the major strains of *E. granulosus* throughout China [16, 43–45]. However, in this study, the G1–G3 isolates displayed distinct nucleotide differences to the reference sequences of G1–G3. Further studies are needed to ascertain the reasons for these differences.

Both the phylogenetic tree and the parsimony network showed that genetic relationships among haplotypes were not associated with geographical divisions, as haplotypes from all regions grouped together. This finding indicates that the regional populations of Southwest China are not fully differentiated from each other. The low pairwise Fst values we found are consistent with this observation of low genetic variation among the three sampling locations. Interestingly, an analogous genetic structure was found for *E. granulosus* in Tibet plateau by Yan et al. [45] using 28 ND1+ATP6 gene haplotypes and in eastern Tibet and Xinjiang by Nakao et al. [46] using 43 CO1 gene haplotypes. These results suggest that the population structure of *E. granulosus s. s.* may be highly uniform throughout China. However, a larger number of samples from more regions would need to be collected and analyzed to confirm this idea.

Overall, the haplotypes showed low nucleotide (Pi < 5%) and high haplotype diversities (Hd > 0.5) consistent with the hypothesis of sudden demographic expansion [47]. This hypothesis was supported by the finding of large negative Fu's Fs and Tajima's *D* values and the single peak observed in the mismatch distribution [48]. This proposed demographic expansion may be caused by the migration of large numbers of host species (sheep, yaks, and dogs) to new areas in response to environmental change or by the artificial introduction of new hosts.

The *ND2* gene similarity of the 51 *E. granulosus* isolates was found to be high (96.71% to 100%), though no sequence displayed 100% homology with the G1 genotype. The genetic diversity observed among the isolates in this study was significantly higher than that reported in previous studies, which used the *CO1* gene to investigate genetic polymorphisms in *E. granulosus* in China [46]. However, the number of haplotypes identified in the previous study was much higher than that identified in the current study (43 versus 19). These differences are likely to be a consequence of the faster evolution rate of the *ND2* gene than the *CO1* gene of *E. granulosus*. Nevertheless, the high degree of genetic diversity in *E. granulosus* in this region revealed by all the studies indicates that the populations are genetically robust and likely to survive and spread.

In summary, our study provides the basic information about genetic diversity analysis of a wider range of isolates from different regions of Southwest China in order to understand in detail the genetic structure of *E. granulosus *populations and transmission dynamics of echinococcosis in these regions. Having the useful information, focus should now be directed to strengthen disease surveillance in these regions and improve diagnostic and prevention methods, in addition to developing a robust control strategy.

## Figures and Tables

**Table 1:** Number of *Echinococcus granulosus* isolates obtained from the three intermediate host species in the three localities.

Locality	Host			Total
	Sheep	Humans	Yaks	
Qinghai	35			35
Sichuan		7	2	9
Tibet	5		2	7

**Table 2:** *ND2* gene sequence information for *Echinococcus* species.

Species (genotype)	Location	Host	Database accession number
*E. granulosus *(G1)	Britain	Sheep	AF297617
*E. equinus* (G4)	Britain	Horse	AF346403
*E. ortleppi* (G5)	Argentina	Buffalo	AB235846
*E. canadensis* (G6)	Kazakhstan	Camel	AB208063
*E. canadensis* (G7)	Poland	Pig	AB235847
*E. canadensis* (G8)	America	Moose	AB235848
*E. multilocularis *	Japan	Hamster	AB018440

**Table 3:** Multiple alignments of the 19 mtDNA haplotypes found in this study. Haplotype identification (Hp), GenBank accession number, number of isolates (*n*), percentage (%), and nucleotide positions (Np) of point mutations are reported.

Haplotypes	h1	h2	h3	h4	h5	h6	h7	h8	h9	h10	h11	h12	h13	h14	h15	h16	h17	h18	h19
*n*	6	14	2	5	3	3	3	1	1	1	1	1	1	2	1	1	1	2	2
%	11.8	27.5	3.9	9.8	5.9	5.9	5.9	2.0	2.0	2.0	2.0	2.0	2.0	3.9	2.0	2.0	2.0	3.9	3.9
NP																			
80^b^								C^1^											
90^c^																G^1^			
152^b^																		T^2^	
153^c^																		G^2^	
155^b^																		T^2^	
156^c^																		C^1^	
157^a^																		T^1^	
159^c^																		G^2^	
162^c^																		T^2^	
164^b^																		T^2^	
167^a^																		T^2^	
227^b^															C^1^				
262^a^									C^1^										
309^c^								G^1^											
339^c^																			G^1^
357^c^			C^1^																
362^b^	C^1^																C^1^		
385^a^																			
414^c^	A^2^																		
434^b^																C^1^			
457^a^												A^1^							
460^a^													G^1^						
470^b^																A^1^			
471^c^									T^1^		T^1^				T^1^				
475^a^				C^1^															
487^a^								G^2^									C^1^		
546^c^															A^1^				
552^a^										C^1^									
578^b^																	G^1^		
601^a^								C^1^											
613^a^					G^2^														
618^c^												A^1^							
621^c^						A^1^												A^1^	
625^a^												G^1^							
653^b^							G^1^												
700^b^				C^1^															
715^a^	C^1^																		
794^b^			G^2^																
801^c^									C^1^										
808^a^			C^1^																
809^b^			T^1^																
812^b^			G^1^																
813^c^			A^1^																
822^c^			C^1^																
823^a^			G^2^																
834^c^			A^1^																
836^b^			T^2^																
837^c^			G^2^																
840^c^			G^2^																
842^b^			G^1^																
843^c^			A^2^																

^a^First codon position.

^b^Second codon position.

^c^Third codon position.

^1^Transition mutation.

^2^Transversion mutation.

**Table 4:** Diversity and neutrality indices of *E. granulosus sensu stricto* in Southwest China calculated from nucleotide sequences derived from the *ND2* gene.

Locality	Number of samples	Haplotypes	Haplotype diversity	Nucleotide diversity	Pairwise differences	Tajima's *D*	Fu's FS
Qinghai	35	10	0.837 ± 0.043	0.004 ± 0.003	3.775 ± 1.949	−1.695*	−0.212
Sichuan	9	6	0.917 ± 0.072	0.003 ± 0.002	2.556 ± 1.511	−1.422	−1.549
Tibet	7	6	0.952 ± 0.095	0.008 ± 0.005	6.762 ± 3.639	−0.142	−0.550

Southwest China	51	19	0.899 ± 0.029	0.005 ± 0.003	4.141 ± 2.095	−2.187***	−5.472*

*P < 0.05, ***P < 0.01.

**Table 5:** Results of the analysis of molecular variance to examine genetic differences among *E. granulosus *populations.

Source of variation	Degrees of freedom	Sum of squares	Variance components	Percentage of variance	Fst
Interpopulation	2	8.870	0.202 *V*_*a*_	9.270	0.093
Intrapopulation	48	94.688	1.972 *V*_*b*_	90.730	

Total variance	50	103.549	2.174		

*V*_*a*_: variance component of interpopulation; *V*_*b*_: variance component of intrapopulation.

**Table 6:** Pairwise fixation indices of the *E. granulosus* isolates in the three localities calculated from nucleotide sequences derived from the *ND2* gene.

Region	Qinghai	Sichuan
Qinghai		
Sichuan	0.037	
Tibet	0.142*	0.116

*P < 0.05.
